# The impact of hearing loss on cognitive impairment: The mediating role of depressive symptoms and the moderating role of social relationships

**DOI:** 10.3389/fpubh.2023.1149769

**Published:** 2023-04-04

**Authors:** Xia Cao, Qian Liu, Jiali Liu, Bingfang Yang, Jiansong Zhou

**Affiliations:** ^1^Health Management Center, The Third Xiangya Hospital, Central South University, Changsha, China; ^2^Department of Psychology, Hunan Normal University, Changsha, China; ^3^Department of Psychiatry and National Clinical Research Center for Mental Disorders, The Second Xiangya Hospital, Central South University, Changsha, China

**Keywords:** hearing loss, cognitive impairment, depressive symptoms, social relationships, older adults

## Abstract

**Background:**

Given the potentially negative effects of hearing loss on mental health and cognitive function, it is critical to gain a better understanding of the mechanisms underlying the link between hearing loss and cognitive impairment. This study aimed to investigate the moderating effects of social relationships, including their components in the role of depressive symptoms as a mediator between hearing loss and cognitive impairment.

**Methods:**

Cross-sectional analyses were conducted with 8,094 Chinese older adults (aged ≥65 years) from the Chinese Longitudinal Healthy Longevity Survey in 2018. Simple mediation analysis and moderated mediation analysis were conducted to examine the roles of depressive symptoms and social relationships in the association between hearing loss and cognitive impairment.

**Results:**

There is a significant correlation between hearing loss, depressive symptoms, social relationships, and cognitive function. Depressive symptoms partially mediated the association between hearing loss and cognitive function [standardized regression B-coefficient (B) = −0.114; 95% confidence interval (CI): (−0.158, −0.076)]. Social relationships moderated the effect of hearing loss on cognitive function through both path b (depressive symptoms - cognitive function) [*B* = 0.021; 95% CI: (0.008, 0.034)], and path c’ (hearing loss-cognitive function) [*B* = 0.597; 95% CI: (0.463, 0.730)]. Furthermore, social activities and social networks moderated both the direct and indirect effects of moderated mediation. However, there appeared to be no moderated effect of social support for both the direct and indirect paths.

**Conclusion:**

Social relationships moderated both the direct and indirect effects of depressive symptoms on the association between hearing loss and cognitive impairment. These findings shed light on the mechanisms underlying the relationship between hearing loss and cognitive impairment in Chinese older adults. It might be worthwhile to recommend multidimensional health and social interventions aimed at improving mental health and social inclusion among older adults with hearing loss.

## 1. Introduction

Hearing loss is highly prevalent among older adults and can negatively impact many aspects of later life if not addressed or if their personal communication needs are not supported. About two-thirds of American adults over the age of 70 suffer from hearing loss, but less than 20% of them receive treatment (e.g., hearing aids) (1). In China, more than two-thirds of older adults over the age of 60 suffer from hearing loss, and this number of patients with hearing loss is expected to rise as the population ages (2). Growing evidence of an association between age-related hearing loss (ARHL) and dementia justifies the identification of ARHL as a potentially modifiable risk factor and a possible approach to improving clinical outcomes in patients with dementia.

With global aging, the prevalence of dementia is expected to double every 20 years, and the number of dementia patients worldwide is estimated to soar from 57.4 million in 2019 to 152.8 million in 2050 (3). According to a nationally representative survey conducted in China, dementia predominates among individuals aged 60 and older at 6.0%, and cognitive impairment is present at 15.5%, totaling 15.07 million people with dementia and 38.77 million people with cognitive impairment (4). A vast population with dementia and cognitive impairment has become a significant health burden not just in China but around the world, necessitating the adoption of more effective anti-dementia measures. Despite advances in treatments, neurodegenerative diseases have only achieved limited success. Since hearing loss is highly prevalent among older adults and greatly undertreated, investigating its impact on mental health and the potential social-psychological mediating or moderating factors is an appealing and potentially influential strategy for promoting healthy aging.

Depression is common in older adults. Concerningly, one in five older persons with hearing loss report clinically significant depressive symptoms that necessitate treatment, and hearing loss is also connected to the gradual introduction of new depressed symptoms over time (5). Hearing loss is associated with 1.47 higher odds of depression in older adults, according to a systematic review and meta-analysis recently (6). Most frequently, the association between hearing loss and depression has been examined in the context of psychosocial changes as people age (6). Among older adults with hearing loss, social and emotional loneliness are more likely to make them depressed (7). Meanwhile, difficulty in completing daily activities (8), reduced social activity, and weak social support services as contributing factors in this process (9). Depression may be a contributing factor to hearing loss and cognitive decline because of the overlap of their potential neuropathological mechanisms with the aging brain (10).

Additionally, behavioral explanations for the potential causal relationship discussed above have been put forth. These include social exclusion, loneliness, decreased mobility, and difficulties in everyday tasks, all of which raise the likelihood of cognitive impairment (11). Social relationships have been identified as an important factor for the maintenance or promotion of mental health and cognition among older adults (12). Social relationships, which rely on social networks, make it easier to engage in social activity and access social support (13). People who have a high level of the cognitive reserve are typically more likely to participate in social activities (14). Hence, the cognitive reserve may reduce dementia risk. Given the positive effects of close social ties on health behavior, social interaction may influence cognitive outcomes (social control hypothesis) (13). An intriguing alternative theory put out by Adolphs et al. (15) suggests that social relationships may influence cognitive performance across several domains. Social support, for example, may reduce stress and improve memory and executive function (16). However, few detailed investigations on how social relationships affect cognitive function *via* different domains have been conducted. In the context of the above theory, interindividual variability in social activity, social networks, and social support may produce different outcomes. Correspondingly, we included all three critical dimensions of social relationships in our analyses.

Overall, it is uncommon for research to be undertaken where both psychological and social pathways are addressed at the same time to comprehend how hearing loss affects cognition. Therefore, the first aim of this study was to determine whether depressive symptoms have a mediating effect on the relationship between hearing loss and cognitive impairment. Another aim of this study was to determine whether social relationships, including their components, have any moderating effect on the direct and indirect correlations between hearing loss and cognitive impairment (Supplementary Figure S1). Specifically, we proposed the following hypotheses:

*H1*: Depressive symptoms would act as a mediator between hearing loss and cognitive impairment.

*H2*: The direct and indirect correlations between hearing loss and cognitive impairment would be moderated by social relationships, with depressive symptoms acting as a mediator.

*H3*: The direct and indirect correlations between hearing loss and cognitive impairment would be moderated by social activities, social networks, and social support, with depressive symptoms acting as a mediator.

## 2. Methods

### 2.1. Study design and participants

**Figure 1:**
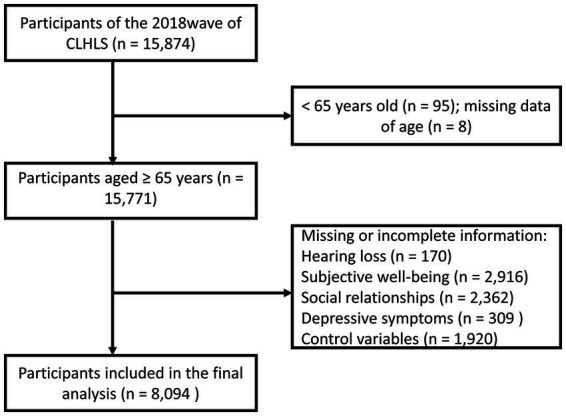
Flow chart of the selection of study participants.

We conducted a cross-sectional analysis of the dataset derived from the eighth wave of the Chinese Longitudinal Healthy Longevity Survey (CLHLS) in 2018, a national representative prospective cohort study of Chinese adults aged 65 and older in major provinces (23 out of 31 provinces) in China. Details of the study participants and methods have been reported elsewhere (17). In the CLHLS 2018, in total, 15,874 face-to-face interviews were conducted using a standard questionnaire. A written informed consent form was obtained from each participant or proxy respondent before the survey. The research has been reviewed and approved by the Research Ethics Committee of Peking University (approval number: IRB00001052-13074). Those younger than 65 years of age (*n* = 95) were excluded from the current analysis. And we restricted our final analysis to 8,094 older adults with completed information on the questions we are concerned about. Details of the screening procedure are described in Figure 1.

Participants with no Mini-Mental State Examination (MMSE) scores, data on frailty, depression, or missing data on social ties were eliminated. If any of the sample’s important variables have missing values, they will be eliminated as well. This analysis comprised 7,525 individuals’ data.

Based on prior research (5, 18, 19) and the design of the CLHLS questionnaire, a set of variables was selected for analysis (Supplementary Table S1).

### 2.2. Dependent variables

#### 2.2.1. Cognitive function

Based on prior research, the Chinese adaptation of the modified Mini-Mental State Examination (MMSE) was utilized in this study to assess cognitive function (17, 20). The MMSE was modified by the CLHLS research team to facilitate older adults’ better understanding and response. It has been widely used in prior studies and has been proven to have good validity and reliability (21–23). Scores ranged from 0 to 30, with a lower score indicating worse cognitive performance. It includes 24 items regarding orientation, attention, registration, calculation, recall, and language. Cognitive impairment was defined as an MMSE score <18, based on previous studies (24, 25). Cronbach’s α coefficient of the MMSE for this study was 0.91. More details about this scale can be found in Supplementary Table S2.

### 2.3. Independent variables

#### 2.3.1. Hearing loss

To assess the participants’ self-perceived hearing status, the following question was asked (without hearing aids): “Do you feel you have hearing difficulty?” The response options were “Yes” (coded as having hearing loss)/“No” (signified not having hearing loss) (25, 26). Self-reported hearing loss was defined in this study as a “Yes” response to self-perceived hearing difficulties.

### 2.4. Mediators

#### 2.4.1. Depressive symptoms

The 10-item Center for Epidemiologic Studies Depression (CES-D-10) was used to assess depressive symptoms, which was a self-reported scale for assessing the symptoms of depression in the past week (27). The CES-D-10 contains 10 items on somatic symptoms, depression impacts, and positive affect. In each item, a score is assigned between 0 and 3 (“rarely” to “almost always”). A total score between 0 and 30, with higher total scores indicating more severe depressive symptoms. A score of 10 or higher indicates possible depression. The CES-D-10 has been validated among older adults in China (27, 28). Cronbach’s α coefficient of the CES-D-10 for this study was 0.87.

### 2.5. Moderator

#### 2.5.1. Social relationships

According to previous studies (19, 29), social relationships were measured as a composite score based on three subdimensions: social activity (ranging from 0 to 3), social networks (ranging from 0 to 4), and social support (ranging from 0 to 6), with a total score ranging from 0 to 13. Detailed variable codes are presented in Supplementary Table S3.

In the context of Berkman et al.’s framework (30), playing cards/mahjong, participating in organized social activity, and visiting experiences were included in the present study, under the subdomain of social activity (29). The concept of social networks can be described as an individual’s web of connections (13). It was measured based on four objective domains, including marital status, living arrangements, and having relatives or children visiting them. Based on the definitions of social support (30), the relevant entries were collected in our study based on questions about who is available to assist with six common life scenes (29), as described in Supplementary Table S3.

### 2.6. Covariates

As covariates, sociodemographic characteristics, lifestyles, and health status were classified as potentially related factors in previous studies (31).

#### 2.6.1. Sociodemographic characteristics

The sociodemographic characteristics contained age (in years), gender (male, female), education (illiterate, literate), residence (rural, urban), and financial support (insufficient, sufficient).

#### 2.6.2. Lifestyle

According to a recent study (32), a combined lifestyle score, ranging from 10 to 50, was created by summing the dietary pattern score and the daily life habits score. Since the missing proportion of the collected data in this domain was less than 5%, we chose to replace the missing values with the average score of each variable. Eight different dietary groups—including staple foods, fresh fruits and vegetables, meat, fish, sugar, milk, and nuts—had their intake frequency monitored (33). A dietary pattern score is equal to the sum of the scores of all eight food groups ranging from 7 to 38, with higher scores indicating healthier dietary patterns, which were described in previous studies (32–34). As part of the survey, participants were also asked to recall the frequency and amount of tobacco and alcohol consumption, as well as the amount of outdoor exercise they did. Scores for tobacco use and alcohol consumption ranged from 1 to 4 and 1 to 3, respectively. A higher score indicated fewer daily smoking or drinking sessions (32). The participants were asked to rate how frequently they engaged in outdoor activities, with a score ranging from 1 to 5 in ascending order, depending on how frequently they did so. Across all daily life habits, the score ranged from 3 to 12 (32). Detailed variable codes are presented in Supplementary Table S4.

#### 2.6.3. Health status

In this study, health status mainly involves the ability of daily living (ADL) and chronic diseases. The ADL scale was used to evaluate functional ability, which includes six domains: bathing, dressing, eating, toileting, continence, and indoor transfer. Scores were assigned based on the independence of individuals in completing each of the above actions: 1 = complete dependency, 2 = partial independence, and 3 = complete independence. After adding six items, the ADL score ranged from 6 to 18. Responses with higher scores indicated greater independence and functional ability. Cronbach’s α coefficient of the ADL for this study was 0.86. Detailed variable codes are presented in Supplementary Table S5. For the health status, the CLHLS adopted a list of 13 chronic diseases or conditions (e.g., hypertension, diabetes, heart diseases, stroke, cancers, Parkinson’s disease) to measure comorbidity; an individual was considered to have physical comorbidities (yes or no) if he or she self-reported more than two of these thirteen diseases or conditions at the time of the surveys (35, 36).

### 2.7. Statistical analysis

Summary statistics were reported as the means and standard deviation for numerical variables and as the frequency for categorical variables. Chi-square tests were employed to examine proportional differences, while t-tests were performed to compute mean differences. Pearson correlation analysis was performed to explore the linear relationships between all the variables. PROCESS SPSS macro (Hayes, A.F., Lawrence, KS, USA) was employed to examine the moderated mediation model (37). In Hayes PROCESS, the coefficients of the conditional indirect effects and conditional mediator tests are estimated along with the bias-corrected bootstrap confidence intervals. The regression-based, path-analytic framework we employed in our investigation contains Model 4, Model 59, and Model 76 from the SPSS macro-PROCESS; relevant methods can be referred to earlier works (19, 38). The relationship between hearing loss and cognitive impairment was tested with Model 4 by testing whether depressive symptoms were a mediating factor. The effect of mediation was significant if the 95% confidence interval (CI) of the indirect effects did not include 0. We then used Model 59 to examine whether social relationships mediated both direct (path c’: hearing loss-cognition) and indirect effects (path a: hearing loss-depressive symptoms, and path b: depressive symptoms-cognition). A final step investigated whether the components of social relationships had direct and indirect moderating effects on hearing loss and cognitive impairment by using Model 76. In those models, covariates included age, gender, education level, marital status, residence, financial support, lifestyle scores, ADL scores, and physical comorbidities. The moderated mediation model includes all of the above-mentioned control variables, except for residence and financial support. All analyses were conducted in IBM SPSS 24.0. Significance was determined by a *p*-value less than 0.05 (two-sided tests).

## 3. Results

### 3.1. Sample characteristics

We examine the sample characteristics stratified by the cognitive state in Table 1. Among the 8,094 participants (3,654 male, 4,440 female), 2,248 (27.8%) reported cognitive impairment. Those with cognitive impairment were generally older, female, with lower levels of education, were widowed/separated/single, living in a rural area, with insufficient financial support, had lower lifestyle scores, and had more physical comorbidities (*p* < 0.001). Meanwhile, participants with cognitive impairment were more likely to have hearing loss, depressive symptoms, and poorer social relationships (particularly fewer social activities and fewer social networks) (*p* < 0.001). Neither group had significant differences in ADL scores (*p* > 0.05).

**Table 1:** Characteristics of the sample stratified by cognitive status.

Characteristics	Total Mean ± SD or N (%)	Normal cognition Mean ± SD or N (%)	Cognitive impairment Mean ± SD or N (%)	*χ^2^* or *t* statistics	*p*-value
N	8,094	5,846	2,248	-	-
Hearing loss	2,781 (34.4)	1,510 (25.8)	1,271 (56.5)	678.93	<0.001
Depressive symptoms	2072 (25.6)	1,292 (22.1)	780 (34.7)	135.28	<0.001
CES-D-10 score	7.20 ± 4.40	6.75 ± 4.15	8.36 ± 4.80	−14.95	<0.001
MMSE score	25.35 ± 5.96	28.19 ± 1.84	16.73 ± 5.86	131.32	<0.001
Social relationships	8.56 ± 1.75	8.81 ± 1.72	7.90 ± 1.65	21.65	<0.001
Social activity	0.95 ± 0.83	1.09 ± 0.83	0.57 ± 0.70	28.98	<0.001
Social network	2.69 ± 0.92	2.83 ± 0.90	2.33 ± 0.87	22.91	<0.001
Social support	4.92 ± 1.25	4.89 ± 1.26	5.00 ± 1.19	−3.63	<0.001
Age (years)	83.43 ± 11.25	80.49 ± 10.24	90.06 ± 10.11	−41.96	<0.001
**Gender**					
Male	3,654 (45.1)	2,927 (50.1)	727 (32.3)	206.07	<0.001
Female	4,440 (54.9)	2,919 (49.9)	1,521 (67.7)		
**Education**					
Illiterate	3,657 (45.2)	2059 (35.2)	1,598 (71.1)	843.22	<0.001
Literate	4,437 (54.8)	3,787 (64.8)	650 (28.9)		
**Marital status**					
Married	3,787 (46.8)	3,210 (45.1)	577 (25.7)	557.98	<0.001
Widowed/separated/single	4,306 (53.2)	2,635 (54.9)	1,671 (74.3)		
**Residence**					
Rural	2,300 (28.4)	4,046 (69.2)	1748 (77.8)	58.33	<0.001
Urban	5,794 (71.6)	1800 (30.8)	500 (22.2)		
**Financial support**					
Insufficient	1,039 (12.8)	688 (11.8)	351 (15.6)	21.46	<0.001
Sufficient	7,055 (87.2)	5,158 (88.2)	1897 (84.4)		
Lifestyle score	29.93 ± 4.75	30.37 ± 4.80	28.77 ± 4.39	13.50	<0.001
ADL	16.82 ± 2.34	16.80 ± 2.37	16.80 ± 2.27	−1.590	0.112
**Physical comorbidities**					
Yes	2,611 (32.3)	1800 (30.8)	811 (36.1)	20.61	<0.001
No	5,483 (67.7)	4,046 (69.2)	1,437 (63.9)		

Comparison was performed using the t-test or Chi-square test. SD, standard deviation. CES-D-10, the 10-item Center for Epidemiologic Studies Depression. MMSE, Mini-Mental State Examination. ADL, the activities of daily living.Cognitive impairment was defined as an MMSE score < 24.

### 3.2. The correlation between the study variables

As shown in Table 2, regarding the bivariate correlations, significant associations were found between all study variables, including hearing loss, cognitive function, depressive symptoms, and social relationships. Correlation coefficients did not show evidence of severe multicollinearity, and testing of variance inflation factors confirmed that there was no concern with multicollinearity in the data. Based on these results, further studies may be justified to investigate the moderated mediation effects. In terms of specific components of social relationships, the three domains all showed similar correlations (*p* < 0.001).

**Table 2:** Correlations for the study variables.

	1	2	3	4	5	6	7	8	9	10	11	12	13	14	15
1. Hearing loss	–														
2. Cognitive function	−0.368^**^	–													
3. Depression	−0.213^**^	0.122^**^	–												
4. Social relationships	0.284^**^	−0.173^**^	−0.122^**^	–											
5. Social activity	0.356^**^	−0.209^**^	−0.139^**^	0.560^**^	–										
6. Social network	0.302^**^	−0.209^**^	−0.153^**^	0.529^**^	0.186^**^	–									
7. Social support	−0.060^**^	0.050^**^	0.034^**^	0.643^**^	−0.013	−0.119^**^	–								
8. Age	−0.513^**^	0.426^**^	0.100^**^	−0.309^**^	−0.362^**^	−0.473^**^	0.155^**^	–							
9. Gender	−0.184^**^	0.001	0.112^**^	−0.069^**^	−0.096^**^	−0.209^**^	0.121^**^	0.107^**^	–						
10. Education	0.370^**^	−0.176^**^	−0.146^**^	0.154^**^	0.228^**^	0.264^**^	−0.130^**^	−0.362^**^	−0.393^**^	–					
11. Marital status	0.331^**^	−0.247^**^	−0.120^**^	0.290^**^	0.192^**^	0.677^**^	−0.220^**^	−0.539^**^	−0.295^**^	0.316^**^	–				
12. Residence	−0.079^**^	0.022^**^	0.088^**^	0.079^**^	−0.061^**^	0.005	0.147^**^	−0.031^**^	0.039^**^	−0.243^**^	−0.014^**^	–			
13. Financial support	−0.053^**^	0.016	0.235^**^	−0.035^**^	−0.045^**^	−0.023^**^	−0.002	−0.043^**^	0.013	−0.066^**^	0.009	0.121^**^	–		
14. Lifestyle score	0.174^**^	−0.074^**^	0.188^**^	0.103^**^	0.176^**^	0.095^**^	−0.041^**^	−0.136^**^	0.046^**^	0.187^**^	0.081^**^	−0.351^**^	−0.122^**^	–	
15. ADL	−0.034^**^	−0.001	−0.010	0.005	0.007	−0.014	0.013	0.006	−0.007	−0.016	−0.012	0.027^*^	−0.016	0.007	–
16. Physical comorbidities	0.058^**^	0.021	0.087^**^	0.000	0.025^*^	0.035^**^	−0.043^**^	−0.072^**^	0.011	0.090^**^	0.041^**^	−0.198^**^	0.001	0.165^**^	−0.070^**^

ADL, the activities of daily living. ^*^p < 0.05. ^**^p < 0.01.

### 3.3. The association between hearing loss and cognitive function

As shown in Table 3, Model 1 focuses on the relationship between hearing loss and cognitive function. Results indicated that hearing loss was significantly associated with cognitive function [*B* = −4.620, 95% CI: (−4.880, −4.361)]. Model 2 explored the relationship between hearing loss and cognitive function when adjusting for sociodemographic characteristics, lifestyles, and health status. Hearing loss was significantly associated with cognitive function [*B* = −2.249, 95% CI: (−2.505, −1.994)]. Age [*B* = −0.186, 95% CI: (−0.199, −0.173)], gender [*B* = −1.046, 95% CI: (−1.295, −0.797)], financial support [*B* = −0.752, 95% CI: (−1.084, −0.419)], and ADL [*B* = −0.065, 95% CI: (−0.112, −0.018)] were negatively associated with cognitive function. Education [*B* = 1.793, 95% CI: (1.526, 2.060)] and total lifestyle score [*B* = 0.098, 95% CI: (0.073, 0.124)] were positively associated with cognitive function. The significance and direction of the correlation coefficients between hearing loss and cognitive function did not change from Model 1 to Model 2, indicating that hearing loss is a significant predictor of cognitive function.

**Table 3:** The association between hearing loss and cognitive function.

	Model 1					Model 2				
	*B*	SE	*p*-value	LLCI	ULCI	*B*	SE	*p*-value	LLCI	ULCI
Hearing loss	−4.620	0.132	<0.001	−4.880	−4.361	−2.249	0.130	<0.001	−2.505	−1.994
Age						−0.186	0.007	<0.001	−0.199	−0.173
Gender						−1.046	0.127	<0.001	−1.295	−0.797
Education						1.793	0.136	<0.001	1.526	2.060
Marital status						0.219	0.138	0.113	−0.052	0.490
Residence						−0.155	0.138	0.259	−0.425	0.115
Financial support						−0.752	0.169	<0.001	−1.084	−0.419
Lifestyle score						0.098	0.013	<0.001	0.073	0.124
ADL						−0.065	0.024	0.006	−0.112	−0.018
Physical comorbidities						0.074	0.121	0.542	−0.163	0.311

*B*, standardized regression B-coefficient; SE, standard error; LLCI, lower limit confidence interval; ULCI, upper limit confidence interval. ADL, the activities of daily living. Model 1 investigated the association between hearing loss and cognitive function.Model 2 investigated the association between hearing loss and cognitive function when adjusting for covariates.

## 4. The mediating role of depressive symptoms in the association between hearing loss and cognitive function

To further elucidate the mechanisms underlying the association between hearing loss and cognitive function, we examined the mediating role of depressive symptoms. Hearing loss and depressive symptoms were positively correlated [*B* = 0.745, 95% CI: (0.527, 0.964)] in a simple mediation model (Model 4), as shown in Table 4. There was an inverse relation between depressive symptoms and cognitive function [*B* = −0.153, 95% CI: (−0.179, −0.127)]. Hearing loss was also associated with cognitive function [*B* = −2.135, 95% CI: (−2.389, −1.881)]. Furthermore, we found that hearing loss was associated with depressive symptoms, which had a significant indirect effect on cognitive function [*B* = −0.114; 95% CI: (−0.158, −0.076)], accounting for 5.07% of the total effect.

**Table 4:** Testing the mediating effect of hearing loss on cognitive function.

Variable	Path	*B*	SE	LLCI	ULCI
Total effect	Hearing loss-Cognition	−2.249	0.130	−2.505	−1.994
Direct effect	Hearing loss-Depression	0.745	0.111	0.527	0.964
	Depression-Cognition	−0.153	0.013	−0.179	−0.127
	Hearing loss-Cognition	−2.135	0.129	−2.389	−1.881
Indirect effect	Hearing loss-depression-Cognition	−0.114	0.021	−0.158	−0.076

*B*, standardized regression B-coefficient; SE, standard error; LLCI, lower limit confidence interval; ULCI, upper limit confidence interval.The mediation model was controlled for covariates (age, gender, education level, marital status, residence, financial support, lifestyle scores, ADL scores, and physical comorbidities).

### 4.1. Moderated mediation effects of hearing loss on cognitive function

**Figure 2:**
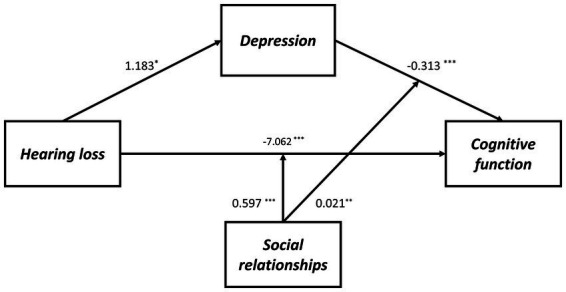
The final moderated mediation model: social relationships as moderator of the mediation model of depression between hearing loss and cognitive function (Andrew Hayes’s mediation-moderation Model 15, ^*^p < 0.05; ^**^p < 0.01; ^***^p < 0.001). The moderated mediation model was controlled for covariates (age, gender, education level, marital status, lifestyle scores, ADL scores, and physical comorbidities).

**Figure 3:**
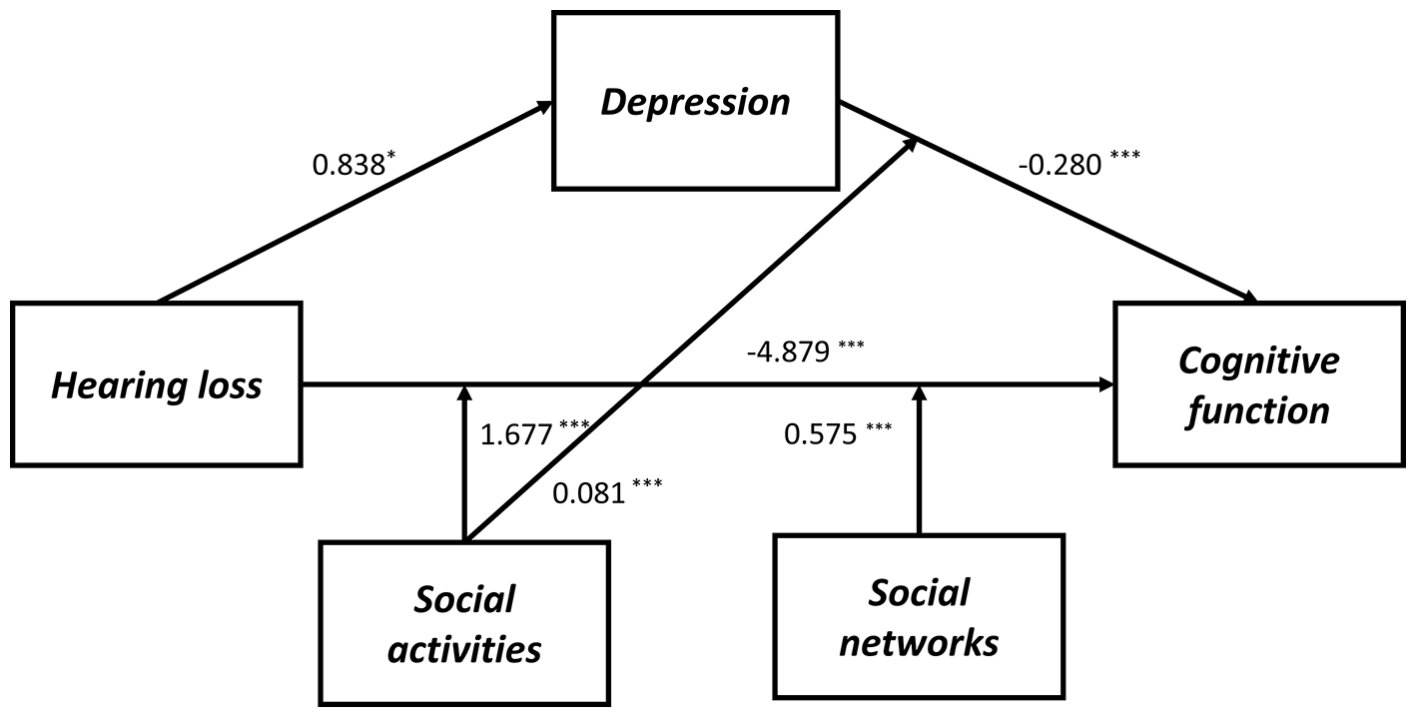
The final moderated mediation model: social activities and social networks as moderators of the mediation model of depression between hearing loss and cognitive function (Andrew Hayes’s mediation-moderation Model 76, ^*^p < 0.05; ^***^p < 0.001). The moderated mediation model was controlled for covariates (age, gender, education level, marital status, lifestyle scores, ADL scores, and physical comorbidities).

As shown in Supplementary Table S6, Model 59 was used to test the proposed moderated mediation model, which indicated that depressive symptoms are not affected significantly by the interaction between hearing loss and social relationships (*B* = −0.056, *p* = 0.339). However, for cognitive function, an interaction effect was observed between depressive symptoms and social relationships (*B* = 0.021, *p* = 0.002), as well as between hearing loss and social relationships (*B* = 0.597, *p* < 0.001). Therefore, the hypothesized model has been modified by removing social relationships as a moderating factor on path a (Figure 2). As a result, in both path b and path c’ of this model, social relationships moderated the effect of hearing loss on cognitive function, as shown in Table 5 and Figure 3.

**Table 5:** Testing the moderated mediating effect of hearing loss on cognitive function by depression and social relationships.

	Depression					Cognitive function				
	*B*	SE	*p*-value	LLCI	ULCI	*B*	SE	*p*-value	LLCI	ULCI
Hearing loss (X)	0.700	0.112	<0.001	0.481	0.920	−1.954	0.128	<0.001	−2.206	−1.702
Depression (M)	–	–	–	–	–	−0.136	0.013	<0.001	−0.162	−0.109
social relationships (W)	−0.177	0.029	<0.001	−0.233	−0.120	0.394	0.033	<0.001	0.329	0.459
XxW	––	–	–	–		0.597	0.068	<0.001	0.463	0.730
MxW	–	–	–	–	–	0.021	0.007	0.002	0.008	0.034

*B*, standardized regression B-coefficient; SE, standard error; LLCI, lower limit confidence interval; ULCI, upper limit confidence interval; X, independent variable; Y, dependent variable; M, mediator.

The moderated mediation model was controlled for covariates (age, gender, education level, marital status, lifestyle scores, ADL scores, and physical comorbidities).

**Figure 4:**
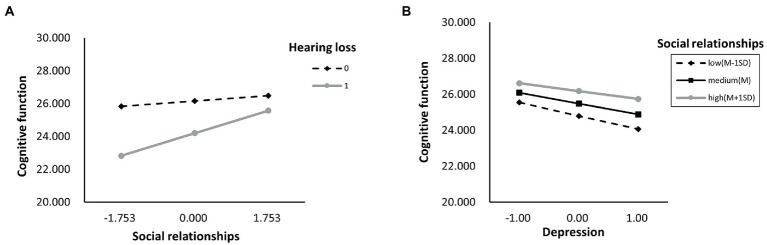
The simple plot of path **A** and **B** indicating the relationship between hearing loss, depressive symptoms, and cognitive impairment among different levels of social relationships.

**Figure 5:**
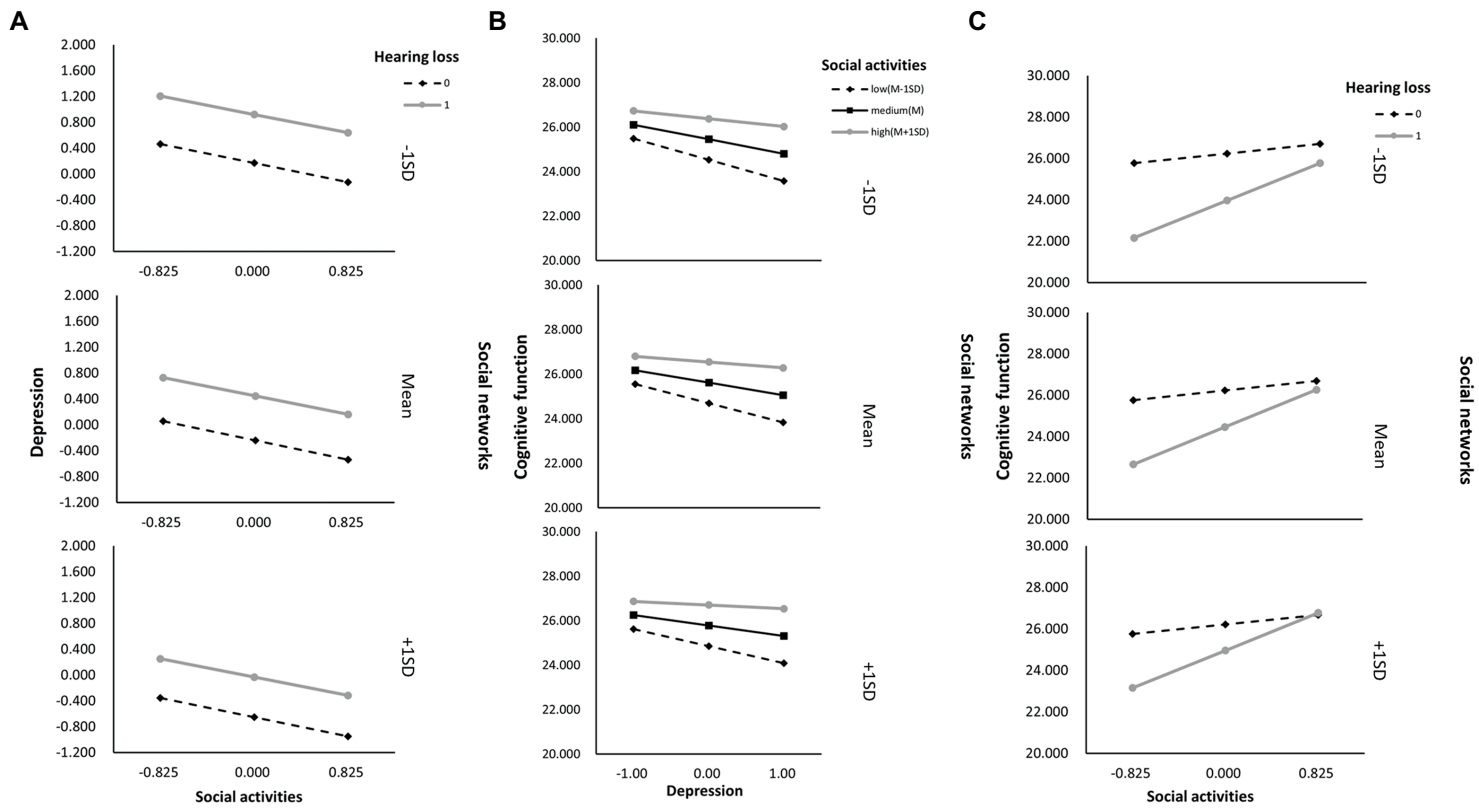
The simple plot of path **A**, **B**, and **C** indicating the relationship between hearing loss, depressive symptoms, and cognitive impairment among different levels of social activities and social networks groups.

As shown in Supplementary Table S7, we also determined whether the effect was moderated by social activity and social networks simultaneously *via* Model 76. As a result, through paths a (hearing loss x social activity: *B* = 1.628, *p* < 0.001), b (depressive symptoms x social activity: *B* = 0.082, *p* < 0.001), and c’ (hearing loss x social activity: *B* = 1.628, *p* < 0.001), social activity moderated the impact of hearing loss on cognitive function. While through both path a (hearing loss x social networks: *B* = 1.628, *p* < 0.001) and path c’ (hearing loss x social networks: *B* = 0.552, *p* = 0.001), social networks moderated the impact of hearing loss on cognitive function. Moreover, the direct and indirect effects of hearing loss on cognition were tested using Model 59, but neither direct nor indirect paths appeared to be moderated by social support (Supplementary Table S8). According to Table 6, the association between hearing loss and cognitive function was negatively moderated by social relationships at any level. A similar effect was observed for social activity and social networks, except both of them, had higher than one standard deviation (Supplementary Table S9). In addition, the simple slope analysis showed that if the social relationships were below (*β* = −3.000, *p* < 0.001) or above (*β* = −0.908, *p* < 0.001) one standard deviation, participants with hearing loss had lower cognitive function than participants with normal hearing (Figure 4A). Moreover, the cognitive function score decreased significantly as the CES-D-10 score increased, whether social relationships were below (*β* = −0.137, *p* < 0.001) or above (*β* = −0.060, *p* < 0.001) one standard deviation (Figure 4B). As well, when social activity and social network were lower, coupled with hearing loss or high CES-D-10 scores, the MMSE score was lower (Figure 5).

**Table 6:** Conditional indirect effects of hearing loss on cognitive function.

Social relationships	*B*	SE	LLCI	ULCI
−1-SD	−0.137	0.033	−0.206	−0.078
Mean	−0.095	0.018	−0.134	−0.062
−1 + SD	−0.060	0.019	−0.099	−0.027

*B*, standardized regression B-coefficient; SE, standard error; LLCI, lower limit confidence interval; ULCI, upper limit confidence interval.

## 5. Discussion

In recent years, there has been an increase in empirical support for the adverse effect of hearing loss on older adults’ cognitive function (39, 40). Although hearing loss has been identified as one of the most important modifiable risk factors for dementia and cognitive decline, no causal relationship has been found between these two conditions (41). In addition, there is a lack of knowledge of the moderated mediation mechanisms underlying this association, particularly for older adults who are more susceptible to the influences of hearing loss. To fill this gap, we chose the representative older adults from the CLHLS database, since the included older adults aged 65 and older are known to be susceptible to hearing loss, as well as exhibiting elevated levels of depressive symptoms and cognitive impairment (25), with a diverse range of observed variables. Using the moderated mediation model, we found that depressive symptoms may partially mediate the association between hearing loss and cognitive impairment in older adults. In addition, the association between hearing loss and cognitive impairment was negatively moderated by social relationships at any level. Moreover, social activity and social networks moderated the direct and indirect path of hearing loss to cognitive impairment, but in the case of social support, the moderation effect was non-significant. In summary, this study sheds light on the impact of hearing loss on cognitive function and its internal social-psychological mediating mechanism among Chinese older adults. The prevalence of cognitive impairment and hearing loss in this study was 27.8 and 34.4%, respectively, which is in agreement with previously reported prevalence levels among older adults (42). Similar to Zhang et al. (19), as shown in our research, a variety of factors may contribute to cognitive impairment, including gender, age, the absence of a spouse, illiterate, living in rural, insufficient financial support, lower lifestyle score, comorbidities, poor social relationships and with depression. We anticipate that identifying the interconnectivity of these risk variables sheds some light on the mechanism of connection between hearing loss and cognitive impairment and that depressive symptoms play an essential part in both, as has piqued our attention. That the identification of the interconnection of these risk factors sheds some light on the mechanism of connection between hearing loss and cognitive impairment, and depressive symptoms play an important role in both, which has piqued our curiosity. These findings serve as starting points for us to identify possible confounders and covariates.

### 5.1. The mediating role of depressive symptoms

In older adults with hearing impairment, depressive symptoms may contribute to additional cognitive problems beyond those associated with their hearing loss (43). According to cognitive theories of depression (44) and previous empirical studies (45, 46), our results indicated that hearing loss could impair older adults’ cognition through depressive symptoms mediation. As reported by Danielle et al., individuals with clinically meaningful hearing loss at levels that may impair communication capability are at risk for accelerated cognitive decline and incident dementia, particularly those who acquire clinically significant depressive symptoms (46). According to cognitive theories of depression, since the significant impact of hearing and communicating effectively on quality of life and behavior, it is possible that additional depressive symptoms may develop. Meanwhile, the onset of a prolonged event—especially if it lasts longer than an acute event—can exacerbate psychosocial or neuropsychological buffers, accelerating cognitive decline among those with hearing loss. Thus, the study emphasizes the role of depression among older adults’ poor physical and social function, as well as their poor mental health.

Furthermore, this mediation model involves several stages that need to be discussed. As for the first stage (i.e., hearing loss → depressive symptoms), the results of our study showed that exposure to hearing loss increased the risk of depressive symptoms among older adults. Hearing loss is associated with depressive symptoms in older adults linked to changes in psychosocial experiences and declines in cortical activity (5, 47, 48). On the one hand, hearing loss is related to higher social and emotional isolation in older adults and may become a chronic stressor if left untreated, contributing to the growth of depressive symptoms as an additional stressor (49). As explained by the stress appraisal theory by Lazarus and Folkman, adaptation fails when stressful situations are perceived as threatening, challenging, or harmful, overriding one’s capacity to cope (50). On the other hand, there is also evidence that neuropathological mechanisms associated with auditory perception and mood regulation may contribute to hearing loss and depressive symptoms, with the limbic system (which regulates emotion, reasoning, and planning), the frontal cortex (which regulates emotion, reasoning, and planning), and auditory cortex exhibit similar patterns of reduced activity among older adults with hearing loss or depressive symptoms (10). It appears that hearing loss and depressive symptoms are associated with common neural degeneration in older adults. As for the second stage (i.e., depressive symptoms → cognitive impairment), CESD-10 was negatively associated with MMSE score, consistent with the findings of Zhou et al. (51). It has been shown that cognitive impairment and depressive symptoms may share at least three pathways (52). First, the presence of persistent mood symptoms might impair cognitive function *via* neurobiological pathways. Chronic stress-induced immunological dysregulation may have a direct influence on cognition through cumulative exposure to emotional symptoms (53). Second, mood symptoms may contribute to and aggravate poor health behaviors that negatively affect cognitive performance, including unhealthy diet, inactivity, smoking and substance abuse, and medication used to treat symptoms (54). In addition, disability may contribute to cognitive impairment as well as depressive symptoms in a reciprocal manner. Depressive symptoms affect patients’ ability to engage in cognitively stimulating activities, as well as their participation in the workforce and mental challenges (55). Meanwhile, people with depressive symptoms tend to have fewer social networks than healthy controls, and they are more likely to experience negative interactions and social strain. Moreover, depressive symptoms are more likely to be associated with poverty and socioeconomic factors could also hinder patients’ access to healthy food, safe physical activity areas, and cognitive skills. As a result, older adults suffering from depressive symptoms are more likely to have reduced cognition.

### 5.2. The moderating role of social relationships

Social relationships negatively correlated with depressive symptoms, as expected. The odds of developing depressive symptoms are lower for older adults who have more social relationships. In line with previous empirical studies (56) and the social support theoretical model (57), this finding suggests that good social relationships could benefit older adults by alleviating the level of depressive symptoms. While older adults with long-term hearing loss face a variety of barriers that may hinder their full and effective access to sufficient social resources (58). The present findings indicated that social relationships moderated the association between hearing loss and depressive symptoms. Those who have strong social relationships are less likely to suffer from depressive symptoms when facing hearing loss than those who have weak social relationships, which suggests that good social relationships may buffer the adverse effects of hearing loss on older adults’ mental health. According to the stress-buffering model and main effects model (59), social connections help people cope with stress by providing psychological and material resources. It is believed that stress affects health both by activating physiological systems such as the sympathetic nervous system and the hypothalamic–pituitary–adrenal axis and by promoting behavioral coping responses detrimental to health (smoking, excessive alcohol, lack of sleep, or substance abuse) (59). The main-effect model contends that social connections are advantageous regardless of one’s level of stress (60). For example, an empirical study based on the stimulus-organism-response (S-O-R) framework showed that social media use promotes strong social relationships among hearing-impaired older adults, as well as improving aging cognition and depressive symptoms (61).

In line with previous findings that strong social relationships buffers depression (49) and problematic behavior of older adults with hearing loss (62). However, it was not investigated whether the components of social relationships buffer hearing loss and depressive symptoms in these studies. According to our knowledge, this study is the first to confirm that two components of social relationships-social activity and social network-serve as buffers against the negative effects of hearing loss on depressive symptoms in a representative sample of Chinese older adults simultaneously, and the findings extend previous studies.

Furthermore, hearing loss and cognitive impairment were also moderated by social relationships. Specifically, hearing-impaired older adults with low social relationships exhibited lower cognitive function than those with high social relationships. This result is consistent with previous studies and extends them by demonstrating the buffering effect of social relationships and its specific component in the association between hearing loss and cognitive decline among older adults (49, 63). The observed buffering effect of social relationships in the present study might be attributed to several potential factors. First, higher levels of social activity are related to greater cognitive reserve, resulting in activating and strengthening various neural circuits and behavioral pathways, improving the ability to compensate for adverse structural and functional brain consequences caused by hearing loss and depression (19, 64). The opposite was social isolation correlates with both restructuring and functional changes in the brain’s social network and in brain regions that are related to mentalizing and social interaction, according to the social brain hypothesis (65). Second, social networks may be whittled down more rapidly for people with incident hearing problems, and it may be beneficial for them to use targeted coping strategies and auditory rehabilitation methods to cope with the stressful consequences of external threats by obtaining appropriate coping resources (66). Third, although social support’ moderating role among hearing loss and cognitive impairment did not reach significance in this study and other CLHLS studies (29), increasing evidence suggests that positive social support is strongly associated with successful hearing aid use and mental health improvement (67). Overall, maintaining positive social relationships may lessen the effects of hearing loss or depressive symptoms on cognitive function.

Some limitations should be addressed in this study. Firstly, the cross-sectional design renders causal inferences about the association between hearing loss, depressive symptoms, and cognitive impairment difficult. The causal direction between hearing loss and cognitive impairment and more accurate mediation estimates could be explored in the future with a longitudinal design. Secondly, self-report measures can be prone to bias and distortion. It is therefore essential to use multiple measures such as an in-depth interview or observation of behavior. Thirdly, since lacked relevant details, the frequency, severity, and duration of hearing loss as well as the information on hearing aids were not considered in this study. Future research should verify whether these factors might be involved in the moderated-mediation model among older adults.

Despite the above limitations, there are theoretical and practical implications to our findings. Based on theoretical considerations, the present findings provide an empirical framework for testing depressive symptoms’ mediating role in the association of hearing loss with cognitive impairment, as well as social relationships’ moderating role. As a consequence of this framework, we may better understand how hearing loss is related to cognitive impairment among Chinese older adults. Around the world, we must shift the way we look at the hearing, hearing loss, and how hearing rehabilitation impacts the overall quality of life of older adults. From a practical view, because hearing loss increases the likelihood of depressive symptoms among older adults, families, caregivers, healthcare personnel, and institutions should pay more attention to older adults with hearing loss. In rehabilitative practice, broader consultations should especially involve discussing emotional elements of social interaction with patients and how hearing loss affects cognitive and physical functioning. Meanwhile, the identification of vulnerable individuals is essential to ensuring that prevention and early intervention programs are targeted at them. This moderating mediation model has the potential to facilitate earlier identification, enhance motivation for hearing aid and treatment, as well as reduce stigma. Overall, this could be beneficial for older adults with hearing loss, their families and social circles, the healthcare system, and society as a whole.

## 6. Conclusion

As a whole, we found that depressive symptoms played a partial mediating role in the association between hearing loss and cognitive impairment among a nationally representative sample of Chinese older adults. Furthermore, in addition to social relationships, these two components, i.e., social activity and social network, moderated the association between hearing loss and cognitive impairment. It might be worthwhile to recommend multidimensional health and social interventions aimed at improving mental health and social inclusion among older adults with hearing loss.

## Data availability statement

The datasets presented in this study can be found in online repositories. The names of the repository/repositories and accession number (s) can be found below: https://opendata.pku.edu.cn/dataverse/CHADS.

## Ethics statement

The research has been reviewed and approved by the Research Ethics Committee of Peking University (approval number: IRB00001052-13074). The patients/participants provided their written informed consent to participate in this study.

## Author contributions

XC and JZ conceived the concept and design of the study. XC and QL contributed to data cleaning and analysis. JL and BY contributed to the writing assistance and proofreading of the article. All authors approved the final version of the manuscript.

## Funding

This study was supported in part by a grant from STI2030-Major Projects (2021ZD0200700), the National Natural Science Foundation of China (71804199, 82071543), the Natural Science Foundation of Hunan (2021JJ30037), the Health Commission of Hunan Province (202103091470, 202215025353), and the Hunan Medical Association (HNA202101008).

## Conflict of interest

The authors declare that the research was conducted in the absence of any commercial or financial relationships that could be construed as a potential conflict of interest.

## Publisher’s note

All claims expressed in this article are solely those of the authors and do not necessarily represent those of their affiliated organizations, or those of the publisher, the editors and the reviewers. Any product that may be evaluated in this article, or claim that may be made by its manufacturer, is not guaranteed or endorsed by the publisher.
