# Supplementary figures and images for: The impact of hearing loss on cognitive impairment: The mediating role of depressive symptoms and the moderating role of social relationships
Source: Front Public Health. 2023 Apr 4;11:1149769. doi: 10.3389/fpubh.2023.1149769 (PMC10116415; doi:10.3389/fpubh.2023.1149769)

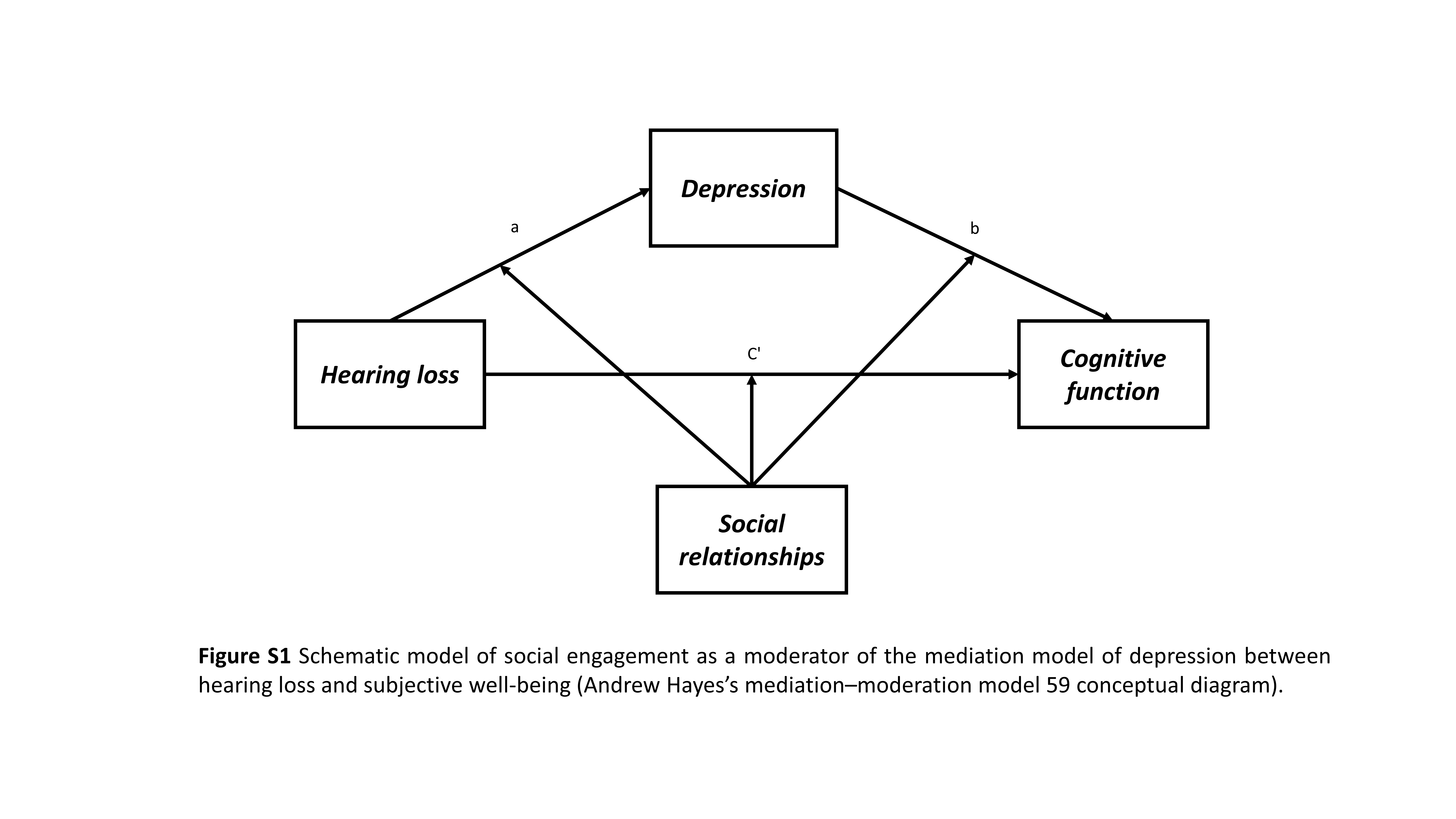

Supplement: Supplementary file 1 [file Image_1.TIF]
