# Supplementary material for: The impact of hearing loss on cognitive impairment: The mediating role of depressive symptoms and the moderating role of social relationships
Source: Front Public Health. 2023 Apr 4;11:1149769. doi: 10.3389/fpubh.2023.1149769 (PMC10116415; doi:10.3389/fpubh.2023.1149769)
Supplement: Supplementary file 2 [file Table_1.docx]

**Supplementary Material**

- **Table S1 Description of study variables**
- **Table S2 The Chinese version of the mini-mental state examination (MMSE) in the CLHLS**
- **Table S3 The coding of social relationships**
- **Table S4 The coding of lifestyle score**

- **Table S5 The coding of the activities of daily living (ADL) score**

- **Table S6 Testing the moderated mediating effect of hearing loss on cognitive function by** **depression and social relationships**

- **Table S7 Testing the moderated mediating effect of hearing loss on cognitive function by depression, social activities and social networks**
- **Table S8 Testing the moderated mediating effect of hearing loss on cognitive function by depression and social support**
- **Table S9 Conditional indirect effects of hearing loss on cognitive function at different levels of social activities and social networks**

**Table S1. Description of study variables**

| Variables | Description |
| --- | --- |
| **Independent variables** |  |
| Hearing loss | 0 = No, 1 = Yes |
| **Dependent variables** |  |
| Cognitive function | MMSE score (Range: 0-30) |
| **Mediator** |  |
| Depressive symptoms | CES-D-10 score (Range: 0-30) |
| **Moderator** |  |
| Social relationships | Social relationships score (Range: 0-13) |
| Social activity | Social activity score (Range: 0-3) |
| Social networks | Social networks score (Range: 0-4) |
| Social support | Social support score (Range: 0-6) |
| **Covariates** |  |
| Age | Validated age of the sample |
| Gender | 0 = Male, 1 = Female |
| Education | 0 =Illiterate, 1 = Literate |
| Residence | 0 = Rural, 1 = Urban |
| Financial support | 0 = Insufficient, 1 = Sufficient |
| Lifestyle | Total lifestyle score (Range: 10-50) |
| ADL | ADL score (Range: 0-20) |
| Physical comorbidities | 0 = No, 1 = Yes |

MMSE, Mini-Mental State Examination. CES-D-10, The 10-item Center for Epidemiologic Studies Depression. ADL, the activities of daily living.

| **Table S2. The Chinese version of the mini-mental state examination in the CLHLS** | | |
| --- | --- | --- |
| **Domains** | **Questions** | **Scores** |
| Orientation | What time of day is it right now (morning, afternoon, evening)? | 1 |
|  | What is the month (Western or Chinese calendar) right now? | 1 |
|  | What is the date (Chinese calendar day and month) of the mid-autumn festival? | 1 |
|  | What is the season right now, spring, summer, fall, winter? | 1 |
|  | What is the name of this district or town? | 1 |
|  | Please name as many kinds of food as possible in 1 minute. | 7 |
| Registration | repeat table, apple and clothes | 3 |
| Attention and calculation | I will ask you to spend 3 dollars from 20 dollars, then you must spend 3 dollars from the number you arrived at and continue to spend 3 dollars until you are asked to stop. | 5 |
|  | Asking the interviewee to copy a figure, in which all the sides and angles are correct | 1 |
| Recall | repeat the three words (in any order) that you heard a little while ago | 3 |
| Language | Naming pen and watch. | 2 |
|  | Repeating the following sentence: “What you plant, what you will get.” | 1 |
|  | The individual is asked to take a paper using right hand, fold it in the middle using both hands, and place the paper on the floor. | 3 |

| **Table S3. The coding of social relationships** | |
| --- | --- |
| **Variable** | **Coding** |
| **Social Relationships (score: 0-13)** |  |
| **Social activities (score: 0-3)** |  |
| Engagement in play cards/mah-jongg | 0=never; 1= almost every day /not daily, but once for a week/not weekly, but at least once for a month/not monthly, but sometimes |
| Engagement in organized activities | 0=never; 1= almost every day /not daily, but once for a week/not weekly, but at least once for a month/not monthly, but sometimes |
| The number of having visiting experience | 0=0; 1= more than 0 |
| **Social networks (score: 0-4)** |  |
| Marital status | 0=widowed/divorced/never married; 1= married |
| Living arrangement | 0=alone; 1=with household member /in an institution |
| Having sibling visiting | 0=nobody; 1= more than 0 |
| Having children visiting | 0=nobody; 1= more than 0 |
| **Social support (score: 0-6)** |  |
| Having people to talk | 0=nobody; 1= spouse/son/daughter/daughter in law/son in law/grandchildren/other relatives/friends/social workers/housekeeper |
| Having people to share thoughts | 0=nobody; 1= spouse/son/daughter/daughter in law/son in law/grandchildren/other relatives/friends/social workers/housekeeper |
| Having people to ask for help | 0=nobody; 1= spouse/son/daughter/daughter in law/son in law/grandchildren/other relatives/friends/social workers/housekeeper |
| Having people to be cared when having sick | 0=nobody; 1=spouse/son/daughter/daughter in law/son in law/son and daughter/grandchildren/other relatives/friends and neighbors/social services/ live-in care giver |
| Financial support from son | 0=0 yuan; 1= more than 0 yuan |
| Financial support from daughter | 0=0 yuan; 1= more than 0 yuan |

| **Table S4. The coding of lifestyle score** | |
| --- | --- |
| **Variable** | **Coding** |
| **Lifestyle score (score: 10-50)** |  |
| **Dietary pattern score (score: 7-38)** |  |
| The staple food pattern (score: 0-5) | 0= did not take corn as staple food; 1-5= took corn according to the quintile ranking of daily consumption  the quintile ranking of daily consumption month/not monthly, but sometimes |
| Fruit intake (score: 1-4) | 1-4= rarely or never/occasionally/quite often/every day or almost/every day  never /not daily, but once for a week/not weekly, but at least once for a month/not monthly, but sometimes |
| Vegetable intake (score: 1-4) | 1-4= rarely or never/occasionally/quite often/every day or almost/every day |
| Fish intake (score: 1-5) | 1-5= rarely or never/occasionally/at least once per month/ at least once per week/almost every day  week /every day |
| Milk intake (score: 1-5) | 1-5= rarely or never/occasionally/at least once per month/ at least once per week/ almost every day  week /every day |
| Nut intake (score: 1-5) | 1-5= rarely or never/occasionally/at least once per month/ at least once per week/almost every day  week /every day |
| Meat intake (score: 1-5) | 1-5= almost every day /at least once per week/ at least once per month/ occasionally/ rarely or never |
| Sugar intake (score: 1-5) | 1-5= almost every day /at least once per week/ at least once per month/ occasionally/ rarely or never |
| **Daily life habit scores (score: 3-12)** |  |
| Tobacco use (score: 1-4) | 1-3=had the third, second, and first tertile amount of smoking times per day; 4= did not smoke  amount of smoking times per day; 1= spouse/so |
| Alcohol consumption (score: 1-3)  consumption | 1-3= daily alcohol consumption in the third tertile/in the middle tertile /in the first tertile  was in the first tertile |
| Performed outdoor activities (score: 1-5) | 1-5=never/not every month but sometimes /not every week but at least once a month/not every day but at least once a week/  but at least once a week/almost every day  but at least once a week (4); not every week, but at least  once a month (3); not every month, but sometimes (2); or  never (1). |
|  | almost every day |

| **Table S5. The coding of the activities of daily living (ADL) score**  **Variable Coding**  **Social Relationships (score: 0-13)**  **Social activities (score: 0-3)**  **Engagement in play cards/mah-jongg 0=never; 1= almost every day /not daily, but once for a week/not weekly, but at least once for a month/not monthly, but sometimes**  **Engagement in organized activities 0=never; 1= almost every day /not daily, but once for a week/not weekly, but at least once for a month/not monthly, but sometimes**  **The number of having visiting experience 0=0; 1= more than 0**  **Social networks (score: 0-4)**  **Marital status 0=widowed/divorced/never married; 1= married**  **Living arrangement 0=alone; 1=with household member /in an institution**  **Having sibling visiting 0=nobody; 1= more than 0**  **Having children visiting 0=nobody; 1= more than 0**  **Social support (score: 0-6)**  **Having people to talk 0=nobody; 1= spouse/son/daughter/daughter in law/son in law/grandchildren/other relatives/friends/social workers/housekeeper**  **Having people to share thoughts 0=nobody; 1= spouse/son/daughter/daughter in law/son in law/grandchildren/other relatives/friends/social workers/housekeeper**  **Having people to ask for help 0=nobody; 1= spouse/son/daughter/daughter in law/son in law/grandchildren/other relatives/friends/social workers/housekeeper**  **Having people to be cared when having sick 0=nobody; 1=spouse/son/daughter/daughter in law/son in law/son and daughter/grandchildren/other relatives/friends and neighbors/social services/ live-in care giver**  **Financial support from son 0=0 yuan; 1= more than 0 yuan**  **Financial support from daughter 0=0 yuan; 1= more than 0 yuan** | |
| --- | --- |
| **Variable** | **Coding** |
| **ADL score (score: 6-18)** |  |
| Bathing | 1 = complete dependency; 2 = partial independence; 3 = complete independence. |
| Dressing | 1 = complete dependency; 2 = partial independence; 3 = complete independence. |
| Eating | 1 = complete dependency; 2 = partial independence; 3 = complete independence. |
| Toileting | 1 = complete dependency; 2 = partial independence; 3 = complete independence. |
| Continence | 1 = complete dependency; 2 = partial independence; 3 = complete independence. |
| Indoor transfer | 1 = complete dependency; 2 = partial independence; 3 = complete independence. |

| **Table S6.** **Testing the moderated mediating effect of hearing loss on cognitive function by depression and social relationships** | | | | | | | | | | | |
| --- | --- | --- | --- | --- | --- | --- | --- | --- | --- | --- | --- |
|  | **Depression** | | | | |  | **Cognitive function** | | | | |
|  | **B** | **SE** | ***P*-value** | **LLCI** | **ULCI** |  | **B** | **SE** | ***P*-value** | **LLCI** | **ULCI** |
| Hearing loss (X) | 0.700 | 0.112 | <0.001 | 0.481 | 0.920 |  | -1.954 | 0.128 | <0.001 | -2.206 | -1.702 |
| Depression (M) | - | - | - | - | - |  | -0.136 | 0.013 | <0.001 | -0.162 | -0.109 |
| Social relationships (W) | -0.177 | 0.029 | <0.001 | -0.233 | -0.120 |  | 0.394 | 0.033 | <0.001 | 0.329 | 0.459 |
| XⅹW | -0.056 | 0.059 | 0.339 | -0.172 | 0.059 |  | 0.597 | 0.068 | <0.001 | 0.463 | 0.730 |
| MⅹW | - | - | - | - | - |  | 0.021 | 0.007 | 0.002 | 0.008 | 0.034 |
| Note: B, standardized regression B-coefficient; SE, standard error; LLCI, lower limit confidence interval; ULCI, upper limit confidence interval; X, independent variable; Y, dependent variable; M, mediator. The moderated mediation model was controlled for covariates (age, gender, education level, marital status, lifestyle scores, ADL scores, and physical comorbidities). | | | | | | | | | | | |

| **Table S7. T****esting the moderated mediating effect of hearing loss on cognitive function by depression, social activities and social networks** | | | | | | | | | | | |
| --- | --- | --- | --- | --- | --- | --- | --- | --- | --- | --- | --- |
|  | **Depression** | | | | |  | **Cognitive function** | | | | |
|  | **B** | **SE** | ***P*-value** | **LLCI** | **ULCI** |  | **B** | **SE** | ***P*-value** | **LLCI** | **ULCI** |
| Hearing loss (X) | 0.686 | 0.112 | <0.001 | 0.465 | 0.906 |  | -1.765 | 0.128 | <0.001 | -2.015 | -1.514 |
| Depression (M) | - | - | - | - | - |  | -0.127 | 0.013 | <0.001 | -0.153 | -0.101 |
| Social activities (W) | -0.354 | 0.063 | <0.001 | -0.477 | -0.231 |  | 1.120 | 0.071 | <0.001 | 0.980 | 1.260 |
| Social networks (Z) | -0.471 | 0.059 | <0.001 | -0.588 | -0.355 |  | 0.178 | 0.068 | 0.009 | 0.045 | 0.310 |
| XⅹW | 0.015 | 0.128 | 0.905 | -0.235 | 0.266 |  | 1.628 | 0.146 | <0.001 | 1.342 | 1.915 |
| XⅹZ | -0.072 | 0.114 | 0.527 | -0.295 | 0.151 |  | 0.552 | 0.130 | <0.001 | 0.298 | 0.807 |
| MⅹW | - | - | - | - | - |  | 0.082 | 0.015 | <0.001 | 0.053 | 0.112 |
| MⅹZ | - | - | - | - | - |  | 0.023 | 0.013 | 0.079 | -0.003 | 0.049 |
| Note: B, unstandardized coefficients; SE, the standard error of indirect effects estimated; LLCI, lower limit confidence interval; ULCI, upper limit confidence interval; X, independent variable; Y, dependent variable; M, mediator. The moderated mediation model was controlled for covariates (age, gender, education level, marital status, lifestyle scores, ADL scores, and physical comorbidities). | | | | | | | | | | | |

| **Table S8. Testing the moderated mediating effect of hearing loss on cognitive function by depression and social** **support** | | | | | | | | | | | |
| --- | --- | --- | --- | --- | --- | --- | --- | --- | --- | --- | --- |
|  | **Depression** | | | | |  | **Cognitive function** | | | | |
|  | **B** | **SE** | ***P*-value** | **LLCI** | **ULCI** |  | **B** | **SE** | ***P*-value** | **LLCI** | **ULCI** |
| Hearing loss (X) | 1.026 | 0.433 | 0.018 | 0.178 | 1.875 |  | -2.019 | 0.504 | <0.001 | -3.008 | -1.031 |
| Depression (M) | - | - | - | - | - |  | -0.138 | 0.049 | 0.005 | -0.233 | -0.042 |
| Social support (W) | 0.032 | 0.047 | 0.488 | -0.059 | 0.123 |  | 0.252 | 0.085 | 0.003 | 0.086 | 0.418 |
| XⅹW | -0.056 | 0.084 | 0.504 | -0.221 | 0.109 |  | -0.021 | 0.098 | 0.834 | -0.213 | 0.172 |
| MⅹW | - | - | - | - | - |  | -0.003 | 0.010 | 0.735 | -0.022 | 0.016 |
| Note: B, unstandardized coefficients; SE, the standard error of indirect effects estimated; LLCI, lower limit confidence interval; ULCI, upper limit confidence interval; X, independent variable; Y, dependent variable; M, mediator. The moderated mediation model was controlled for covariates (age, gender, education level, marital status, lifestyle scores, ADL scores, and physical comorbidities). | | | | | | | | | | | |

| **Table S9. Conditional indirect effects of hearing loss on cognitive function at different levels of social activities and social networks** | | | | | |
| --- | --- | --- | --- | --- | --- |
| **Social activities** | **Social networks** | **B** | **SE** | **LLCI** | **ULCI** |
| -1-SD | -1-SD | -3.616 | 0.184 | -3.977 | -3.254 |
| -1-SD | Mean | -3.108 | 0.167 | -3.435 | -2.781 |
| -1-SD | -1+SD | -2.600 | 0.224 | -3.039 | -2.161 |
| Mean | -1-SD | -2.272 | 0.165 | -2.596 | -1.949 |
| Mean | Mean | -1.765 | 0.128 | -2.015 | -1.514 |
| Mean | -1+SD | -1.257 | 0.185 | -1.619 | -0.895 |
| -1+SD | -1-SD | -0.929 | 0.222 | -1.365 | -0.494 |
| -1+SD | Mean | -0.422 | 0.184 | -0.782 | -0.061 |
| -1+SD | -1+SD | 0.086 | 0.217 | -0.339 | 0.511 |
| Note: B, unstandardized coefficients; SE, the standard error of indirect effects estimated; LLCI, lower limit confidence interval; ULCI, upper limit confidence interval; SD, standard deviation. | | | | | |
